# Adaptive Action Chunking for Robotic Imitation Learning

**DOI:** 10.3390/biomimetics11050316

**Published:** 2026-05-02

**Authors:** Qingpeng Wen, Haomin Zhu, Yuepeng Zhang, Linzhong Xia, Bo Gao, Zhuozhen Li

**Affiliations:** 1 School of Sino-German Robotics, Shenzhen University of Information Technology, Shenzhen 518172, China; 2 Shanghai AI NEV Innovative Platform Co., Ltd., Shanghai 201805, China; 3 State Grid Shanghai Municipal Electric Power Company, Shanghai 200125, China

**Keywords:** robot imitation learning, adaptive action chunking, bimanual manipulation

## Abstract

Action chunking strategies in robot imitation learning struggle to dynamically balance between long-range motion efficiency and short-range operational precision due to their fixed planning horizon. This paper presents an Adaptive Action Chunking framework that enables robots to dynamically predict the optimal action chunk length based on real-time visual context. We design an end-to-end dual-branch network comprising a shared visual encoder, a parallel action prediction head, and a chunk-size prediction head. Experiments on two real-world bimanual robot manipulation tasks (transport-and-place and flip-and-handover) demonstrate that the method autonomously derives two distinct intelligent strategy patterns—phase-aware switching and sustained high-frequency adjustment—in response to task uncertainty. It significantly outperforms fixed-chunk baselines in both success rate and efficiency. Ablation studies confirm that the performance gain stems from the adaptive decision-making mechanism itself.

## 1. Introduction

Robot imitation learning, which enables robots to acquire complex manipulation skills by observing human demonstrations, is a pivotal pathway toward general-purpose intelligent robots [1,2]. In recent years, fueled by advances in deep representation learning [3] and sequence modeling [4], methods that learn action policies directly from high-dimensional visual inputs have achieved remarkable success. Among them, action chunking has emerged as a predominant framework in visual imitation learning [5,6]. It enhances training stability and execution efficiency by decomposing long-horizon tasks into shorter action sequences executed in an open loop [7].

However, prevailing action chunking methods rely on a critical assumption: the planning horizon (i.e., the action chunk length) is fixed [5,8]. While this simplified design is effective for many tasks, it introduces a fundamental constraint: within a single task, the robot cannot dynamically make a trade-off between long-range motion efficiency and short-range operational precision. Specifically, a long chunk favors smooth and efficient long-distance movement but is prone to failure at critical contact phases requiring fine adjustment due to open-loop error accumulation [9]. A short chunk improves control accuracy but results in jerky motions and overall inefficiency [10]. This dilemma is particularly acute in complex, long-horizon manipulation tasks, limiting the robustness and generality of existing methods in real-world scenarios [11].

To resolve this tension, we propose a novel Adaptive Action Chunking framework. The core idea is to move beyond a fixed planning horizon. It enables the robot to dynamically predict the optimal action chunk length based on real-time visual context. This approach is inspired by the principle of meta-learning for adaptive control [12]. To this end, we design an end-to-end dual-branch neural network architecture: a shared Vision Transformer encoder [13] extracts scene features; an action prediction head generates a long future action sequence; and a parallel chunk-size prediction head outputs the optimal discrete chunk length for the current timestep. A deterministic gating module then slices the corresponding sub-sequence from the long action sequence according to the predicted chunk length for robot execution. The model is trained end-to-end by minimizing a weighted sum of the action prediction error and the chunk-size classification error, thereby learning when to adopt which planning horizon.

We systematically evaluate the proposed method on two challenging real-world bimanual robot manipulation tasks. In the bimanual transport-and-place task, the adaptive policy successfully demonstrates intelligent phase-aware switching: employing a long horizon during transport for smooth motion and automatically switching to a short horizon for precision placement. In the more demanding bimanual alternating flip-and-handover task, which involves sustained high-uncertainty contact, the policy exhibits a sustained high-frequency adjustment mode, persistently using the minimum chunk length to maximize closed-loop response frequency. Quantitative results show that our method significantly outperforms all fixed-chunk baselines in both success rate and efficiency on both tasks. Ablation studies further confirm that the performance gain stems primarily from the adaptive decision-making mechanism itself, not merely increased network capacity.

The main contributions of this paper are threefold:

1. We propose a new paradigm of adaptive action chunking. This work is the first to unify long-horizon action generation and dynamic planning-horizon decision-making within an end-to-end imitation learning framework, fundamentally overcoming the inherent limitations of fixed-chunk strategies.
2. We design an efficient dual-branch network architecture. The synergistic design of shared encoding and parallel prediction enables the joint optimization of action and chunk length prediction. Comprehensive ablation studies validate the core value of the adaptive module.
3. We provide systematic empirical validation. Through comprehensive quantitative and qualitative evidence on two complementary complex manipulation tasks, we not only demonstrate the superiority of the method but also reveal its general capability to derive distinct intelligent strategies based on the uncertainty structure of tasks.

It is worth noting that the proposed adaptive action chunking framework is distinct from existing hierarchical and skill-based learning methods that also focus on temporal abstraction. Specifically, traditional hierarchical learning approaches typically decompose complex tasks into a fixed hierarchy of subtasks, with temporal chunking realized through predefined rules or pre-set subgoal divisions. In contrast, skill-based learning methods often rely on pre-defined primitive skills and offline training, requiring prior preparation before adapting to new scenarios. Different from these approaches, the proposed framework achieves adaptive temporal abstraction through a dual-branch neural network that dynamically predicts chunk lengths based on real-time visual observations. It does not require pre-defined subtasks, offline skill pre-training, or fixed chunk rules. Instead, the chunk length is adaptively adjusted according to the real-time context of the manipulation task (e.g., changes in object position, task complexity), integrating seamlessly with the online imitation learning process. This design enables the framework to balance motion efficiency and operational precision in a more flexible manner, which is difficult to achieve with traditional methods that rely on fixed or pre-defined temporal divisions.

## 2. Related Works

Research in robot imitation learning aims to acquire robotic skills in a data-driven manner, and its progress has laid the foundation for our work. This chapter reviews related work from three perspectives: imitation learning frameworks, action chunking techniques, and adaptive decision-making methods, clarifying the positioning and innovation of our work.

### 2.1. Imitation Learning Frameworks

Imitation learning enables robots to learn policies by observing expert demonstrations. Behavioral Cloning, as one of its main paradigms, learns a policy by minimizing the difference between the agent’s and the expert’s actions in a supervised manner [14]. Early work focused on learning from low-dimensional state features [15], while the rise of deep learning has made it possible to learn end-to-end policies directly from high-dimensional visual inputs, achieving remarkable success [16]. However, standard behavioral cloning faces generalization challenges in long-horizon tasks due to the compounding error problem [17]. To address this, researchers have proposed methods combining Inverse Reinforcement Learning [18] or introducing adversarial training [19] to infer the underlying reward function from data or improve policy robustness. These frameworks show potential in complex tasks but often have higher requirements on data quality or training stability. Our work builds upon the simplicity of behavioral cloning but introduces a novel temporal abstraction mechanism specifically to address its long-horizon planning difficulties.

### 2.2. Action Chunking and Sequence Modeling

To improve temporal consistency and execution efficiency in long-horizon tasks, action chunking has been widely adopted as an effective method for temporal abstraction. The core idea is for the policy to output a short action sequence (chunk) at once, which is then executed open-loop for multiple timesteps, thereby mitigating error drift caused by autoregressive inference [20]. In this direction, the Action Chunking Transformer achieved a breakthrough in directly predicting action sequences from visual inputs by leveraging the powerful sequence modeling capability of the Transformer architecture, becoming one of the predominant frameworks in visual imitation learning [5]. A series of subsequent works have extended this framework, for example, by exploring more expressive action distribution models (e.g., diffusion models) to enhance diversity [21] or improving the encoding of historical information to strengthen temporal understanding [22]. Furthermore, other Transformer-based sequence decision models have also shown potential in robotic planning [23]. Nonetheless, these methods share a critical common premise: the length of the predicted action chunk is preset and fixed [5,8,24]. This simplifying assumption prevents the policy from dynamically adjusting when facing complex task phases requiring different planning granularities, creating an inherent trade-off between motion efficiency and operational precision [25]. Our research directly targets this limitation, aiming to transform the chunk length from a static hyperparameter into a variable that the policy can dynamically decide based on context.

### 2.3. Adaptive and Hierarchical Decision-Making Methods

Enabling agents to adapt to environmental changes or task demands is a core goal in robotics. Related research provides conceptual inspiration for our adaptive decision module.

Hierarchical Planning and Skill Learning: These methods manage complexity by decomposing tasks into high-level planning and low-level skill execution. The high level may generate subgoal sequences [26] or select macro-actions from a skill library [27], while the low level is responsible for their implementation. However, their performance often relies on carefully designed skill priors or complex hierarchical structure learning, and the switching between levels is often insufficiently flexible.

Meta-Learning and Parameter Adaptation: Meta-learning aims to enable models to quickly adapt to new tasks. Its “learning to learn” paradigm provides a methodological basis for adaptive adjustment [12,28]. In control, adaptive control methods can adjust controller parameters online to cope with uncertainties in system dynamics [29]. Furthermore, some works have explored conditioning policies to dynamically generate network parameters based on input [30]. Not only is meta-learning applicable to fast task adaptation, but its principles can also extend to dynamically tuning model hyperparameters [31], which aligns with our motivation for adjusting the planning horizon as a meta-parameter. These methods achieve adaptation at different levels. However, they do not treat planning horizon length as a core adjustable meta-parameter. This parameter strongly influences the efficiency and precision of sequential decision-making. Our work is the first to introduce this adaptive decision-making capability into the action chunking framework, enabling the planning horizon to be dynamically and discretely optimized based on real-time perception. To clearly illustrate the differences between our proposed method and previous works, a comprehensive comparison is summarized in Table 1.

## 3. Methods

### 3.1. Problem Formulation

We frame our approach within the context of visual imitation learning for bimanual manipulation. The goal is to learn a policy π that maps the robot’s visual observations to its actions by leveraging a dataset of human demonstrations.

We denote the visual observation at time step t as $o_{t}\epsilon R^{H\times W\times C}$. This observation is an RGB image captured by one or more cameras. The action executed by the robot at time t is represented $a_{t}\epsilon A$, where A is the robot’s action space. In our case, an action $a_{t}$ is a vector comprising the end-effector’s displacement Δx, Δy, Δz; its rotation $\Delta r_{x}$, $\Delta r_{y}$, $\Delta r_{z}$; and the gripper’s open/close command.

The standard objective of imitation learning is to learn a policy π that minimizes the expected difference between the predicted actions and the expert’s actions under the observation distribution of the demonstrations, as shown in Equation (1).

(1) $${min}_{\pi }E_{\left(o_{t},a_{t}^{*}\right)\sim D}\left[L\left(a_{t},a_{t}^{*}\right)\right]$$

where E denotes Expectation, which is used to solve for the minimum expected value of the loss between the predicted action and the expert action under the observation distribution of the expert demonstration dataset D; $a_{t}=\pi (o_{t})$; L is the loss function, which adopts a distance loss function in this paper to measure the deviation degree between the model-predicted action $a_{t}$ and the expert action $a_{t}^{*}$; D is the dataset of expert demonstrations; and $a_{t}^{*}$ is the expert action.

Action Chunking: To improve temporal coherence and stability, the Action Chunking with Transformers (ACT) [5] method learns a policy that predicts a fixed-length sequence of future actions, termed an action chunk, rather than a single action. Formally, the policy is defined as Equation (2).

(2) $$\pi :o_{t}\to \{a_{t},a_{t+1},\ldots ,a_{t+K-1}\}$$

where K is a pre-defined, fixed chunk length. This policy is executed in an autoregressive manner: the entire chunk of K actions is executed open-loop, a new observation $o_{t+K}$ is obtained, and the process repeats.

Core Problem: Limitations of Fixed Chunk Size. While effective, the fixed chunk size K presents a fundamental trade-off:

A small K allows for high-frequency feedback and is suited for precise, contact-rich tasks. However, it results in high computational load and may lead to jerky, myopic motions for long-range movements.

A large K promotes smooth, long-horizon motion planning and reduces computation frequency. However, it is prone to compounding errors and lacks the reactivity needed for fine-grained manipulation.

The key insight of our work is that the optimal chunk size is not static but varies depending on the task context inferred from the visual observation $o_{t}$. Therefore, we introduce the problem of Adaptive Action Chunking, where the goal is to learn a policy that jointly predicts the optimal chunk size $K_{t}$ and the corresponding action sequence, which is formulated as Equation (3).

(3) $$\pi :o_{t}\to (K_{t},\left\{a_{t},a_{t+1},\ldots ,a_{t+K_{t}-1}\right\})$$

where $K_{t}\in [K_{min},K_{max}]$ is a variable dynamically determined at each time step based on the current visual context $o_{t}$. $K_{min}$ is the minimum action chunk length, and $K_{max}$ is the maximum action chunk length, both of which are predefined hyperparameters that limit the value range of the dynamic chunk length $K_{t}$.

### 3.2. Algorithm Overview

To overcome the inherent limitations of a fixed action chunk size, as detailed in Section 3.1, we propose an Adaptive Action Chunking framework. The core of our approach is a dual-branch neural network architecture that dynamically predicts both the optimal chunk length $K_{t}$ and the corresponding action sequence for each control step, based on the current visual observation. This allows the robot to switch between long-horizon planning and short-horizon fine manipulation. It improves both task success rate and execution efficiency.

**Figure 1:**
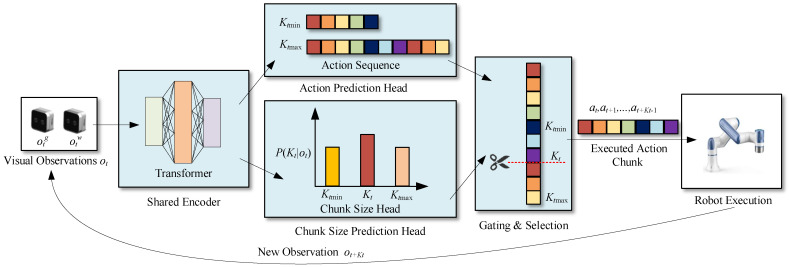
Overview of the adaptive action chunking framework.

Figure 1 provides a high-level overview of the proposed adaptive action chunking pipeline, which operates according to the following procedure:

Multimodal Visual Input: At each timestep t, the system acquires observations from a global camera $o_{t}^{g}$ and a wrist-mounted camera $o_{t}^{w}$. These two image streams provide complementary perceptual information about the task scene.

Shared Feature Extraction: These visual observations are processed by a shared encoder for deep fusion and feature extraction, outputting a unified feature representation rich in semantic scene information.

Dual-Branch Parallel Prediction: This feature representation is subsequently fed into two parallel prediction heads:

The action prediction head regresses a long action sequence spanning a future $K_{max}$ steps: $\left\{a_{t},a_{t+1},\ldots ,a_{t+K_{max}-1}\right\}$. The chunk size prediction head outputs a probability distribution $P(K_{t}|o_{t})$, whose mode represents the dynamically determined optimal chunk length $K_{t}$ based on the current visual context.

Dynamic Chunk Selection and Execution: A gating and selection module uses the output $K_{t}$ from the Chunk Size Head to crop the corresponding $K_{t}$-step action subsequence $\left\{a_{t},a_{t+1},\ldots ,a_{t+K_{t}-1}\right\}$ from the long sequence generated by the Action Head. This action chunk is then executed by the robot in an open-loop manner.

Closed-Loop Autoregressive Control: After executing the action chunk, the system advances to time $t+K_{t}$ and acquires new visual observations $o_{t+K_{t}}^{g}$ and $o_{t+K_{t}}^{w}$. The entire process then repeats, forming a closed-loop, autoregressive control cycle until the task is completed.

In summary, our framework reframes the traditional problem of generating fixed-length action chunks into a joint optimization of action sequences and chunk lengths. This transformation facilitates more intelligent and flexible imitation of complex manipulation tasks. The following sections will elaborate on the detailed design and implementation of the core components within this framework.

### 3.3. Network Architecture

#### 3.3.1. Shared Encoder Design

The shared encoder serves as the visual backbone of our architecture, tasked with processing and fusing multi-view visual observations to extract a unified feature representation. Its design is critical for enabling subsequent prediction heads to make informed decisions based on a comprehensive understanding of the task context.

We employ a standard Vision Transformer (ViT-Base) [13] as the core of our shared encoder, chosen for its powerful capability in capturing global contextual information, which is essential for complex manipulation tasks. The input to the encoder consists of the current frame from the global camera $o_{t}^{g}$ and two wrist-mounted cameras $o_{t}^{w\_left}$ and $o_{t}^{w\_right}$.

Input Preprocessing: Each image is first resized to a fixed resolution of 224 × 224 pixels. The pixel values are then normalized to the range [−1, 1]. We use an early fusion strategy. This fully uses the pre-trained ViT and fuses information from multiple views. Specifically, the normalized images $o_{t}^{g}$, $o_{t}^{w\_left}$, and $o_{t}^{w\_right}$ are concatenated along the channel dimension, forming a combined input tensor of shape [3, 3, 224, 224].

The concatenated input is then partitioned into a sequence of fixed-size patches, which are linearly embedded and augmented with positional information. This sequence is processed by the standard ViT stack of Transformer layers. We utilize the class token ([CLS]) output from the final layer as the global, contextualized feature representation of the multi-view input scene.

The output of the shared encoder is thus a feature vector $f_{t}\in R^{D}$, where D=768 for the ViT-Base configuration. This vector $f_{t}$ encapsulates the fused information from both camera views and serves as the input for the two downstream prediction heads.

#### 3.3.2. Action Prediction Head Design

The Action Prediction Head is responsible for generating a long, unrolled sequence of future motor actions based on the encoded visual context. Its design is critical for producing smooth and kinematically feasible trajectories for the bimanual robot.

Input: The head takes the fused feature representation $f_{t}\in R^{768}$ output by the shared encoder as its input.

Architecture: The head is implemented as a Multi-Layer Perceptron (MLP). The MLP consists of three fully connected (linear) layers with non-linear activation functions interspersed between them to facilitate complex function approximation. The specific design is as follows:

1. A linear layer that expands the feature dimension from 768 to 512.
2. A Rectified Linear Unit (ReLU) activation function that is applied element-wise to introduce non-linearity.
3. A second linear layer that maps the 512-dimensional feature to a 256-dimensional space.
4. Another ReLU activation function.
5. A final linear output layer that projects the 256-dimensional vector to the target output dimension.

Output and Reshaping: The output of the final linear layer is a 1D vector of size $K_{max}\times A$, where

- $K_{max}$ is the pre-defined maximum allowed chunk length.
- A is the dimensionality of the robot’s action space. For our bimanual system, the action space dimensionality is A = 14, comprising 6D end-effector pose increments (Δx, Δy, Δz, $\Delta r_{x}$, $\Delta r_{y}$, $\Delta r_{z}$) for each arm and a 1D gripper open/close command for each gripper.

This flat vector is then reshaped into a 3D tensor of dimensions [B, $K_{max}$, A], where B is the batch size. This tensor represents B batches of proposed action sequences, each sequence containing $K_{max}$ steps, and each step being an A-dimensional action vector. This output is ready for the subsequent gating and selection module.

#### 3.3.3. Chunk Size Prediction Head Design

The Chunk Size Prediction Head constitutes the core innovation of our adaptive framework. It is responsible for dynamically inferring the optimal action chunk length $K_{t}$ based on the current visual context, thereby enabling the robot to autonomously adjust its planning horizon in real-time.

Input: Identical to the Action Prediction Head, this head receives the fused feature representation $f_{t}\in R^{768}$ from the shared encoder as its input.

Architecture: Reflecting the discrete and decision-based nature of its task compared to the continuous action regression, this head is implemented as a more compact Multi-Layer Perceptron (MLP). The design is as follows:

1. A linear layer that maps the input feature dimension from 768 to 256.
2. A Rectified Linear Unit (ReLU) activation function that is applied element-wise.
3. final linear output layer that projects the 256-dimensional feature to a logits vector of size N, where $N=K_{max}-K_{min}+1$ represents the number of all possible discrete chunk length values within the predefined operational range [$K_{min}$, $K_{max}$].

Specifically, $K_{max}$ and $K_{min}$ are determined based on the predefined set of allowable chunk lengths K={5,10,15,20,25,30} (detailed in Section 4.1.4), where $K_{max}=30$ determines the longest action sequence length output by the action prediction head, and $K_{min}=5$ serves as the lower bound of the chunk length. Together, they form the range constraint for the output of the chunk size prediction head, ensuring the predicted chunk length is within a reasonable and task-adaptable scope. Output and Interpretation: The output is a logits vector $l\in R^{N}$. A Softmax function is applied to this vector to convert it into a discrete probability distribution over the possible chunk lengths, following Equation (4).

(4) $$P\left(K_{t}|o_{t}\right)=Softmax\left(l\right)$$

where $P\left(K_{t}|o_{t}\right)$ is the discrete probability distribution of the dynamic chunk length $K_{t}$ under the condition of the current visual observation $o_{t}$. The optimal chunk length $K_{t}$ for the current time step is selected by choosing the value with the highest probability, which is calculated as Equation (5).

(5) $$K_{t}=K\in \underset{\left[K_{\min},K_{\max}\right]}{\mathrm{argmax}}P\left(K|o_{t}\right)$$

This dynamically predicted $K_{t}$ is subsequently passed to the Gating and Selection module (Section 3.3.4), which uses it to crop the corresponding $K_{t}$-step subsequence from the full action sequence generated by the Action Head.

To clarify the generation of ground-truth chunk length, the heuristic rule adopted in this study is based on the real-time complexity of the operation and the rationality of action execution. Specifically, the chunk length is dynamically determined according to the difficulty of the manipulation task: for simple operations (e.g., basic object grasping), a relatively longer chunk length is adopted to improve execution efficiency; for complex operations (e.g., precise positioning and placement), a shorter chunk length is used to ensure operational accuracy. This heuristic design is derived from practical operational experience and the balance between motion efficiency and operational precision, rather than an arbitrary setting.

Regarding the optimality of the heuristic rule, the performance of the model is not significantly affected by small fluctuations in chunk length. Even if the chunk length deviates slightly from the optimal value, the model can still maintain stable operational performance, which indicates that the proposed heuristic method has good robustness and will not lead to performance degradation. In addition, the chunk length generation rule proposed in this study does not rely on complex prior assumptions, and its rationality has been verified through the consistency of experimental results in practical operation scenarios.

#### 3.3.4. Gating and Selection Mechanism

The Gating and Selection module serves as the final, critical stage in our adaptive action chunking pipeline. It is a deterministic, non-learned component that integrates the outputs from the Action Prediction Head and the Chunk Size Prediction Head to produce the executable command for the robot. Its primary function is to implement the dynamic cropping operation based on the predicted chunk length $K_{t}$.

Inputs: The module takes two inputs:

1. The full action sequence $A_{full}\in R^{K_{max}}$ from the Action Prediction Head.
2. The dynamically predicted optimal chunk length $K_{t}\in Z^{+}$ from the Chunk Size Prediction Head, where $K_{t}\in \left[K_{min},K_{max}\right]$.

Operation: The module performs a simple yet crucial selection operation. It acts as a “gate” that selects and crops a contiguous subsequence of length $K_{t}$ starting from the initial time step t from the full sequence $A_{full}$. This can be formally expressed as Equation (6).

(6) $$A_{execute}=A_{full}[:K_{t}]$$

where $A_{execute}\in R^{K_{t}\times A}$ is the resulting executable action chunk. This operation ensures that the robot executes only the immediate $K_{t}$ steps that are deemed most reliable and contextually appropriate by the model.

Upon completion of the $K_{t}$ steps, the system advances its internal clock to time $t+K_{t}$, new visual observations $o_{t+K_{t}}^{g}$ and $o_{t+K_{t}}^{w}$ are acquired, and the entire process—from encoding to gating—repeats. This mechanism forms the foundational bridge between the model’s perceptual predictions and the robot’s physical actions, enabling closed-loop, autoregressive control.

### 3.4. Training Objective and Details

The proposed dual-branch architecture is trained end-to-end on a dataset D of human demonstrations to jointly learn both the action policy and the chunking strategy. This section details the training objectives and hyperparameters.

The total loss function $L_{total}$ is a weighted sum of two component losses, one for each prediction head, as defined in Equation (7).

(7) $$L_{total}=L_{action}+\lambda L_{chunk}$$

where λ is a weighting hyperparameter that balances the scale of the two losses; $L_{action}$ is the Action Prediction Loss, and $L_{chunk}$ is the Chunk Size Prediction Loss. The optimal value of λ is determined based on the characteristics of the dual-branch network and the task requirements. Specifically, λ is set to 1.0 to ensure a balanced optimization of the two component losses: a smaller λ (e.g., 0.1 or 0.5) would underweight the chunk size prediction loss, leading to unstable chunk length decisions where the model tends to output fixed chunk lengths and fails to exhibit effective adaptive behavior. Conversely, a larger λ (e.g., 2.0 or 5.0) would overweigh the chunk size prediction loss, compromising the accuracy of action sequence prediction and further resulting in potential motion jitter or task failure. Setting λ=1.0 enables the dual-branch network to jointly learn precise action sequences and reasonable adaptive chunking strategies, which is consistent with the core design goal of the proposed adaptive action chunking framework. We employ a Mean Squared Error (MSE) loss to regress the predicted action sequences against the ground-truth expert actions. For a predicted sequence ${\hat{A}}_{full}$ and the corresponding expert sequence $A_{full}^{*}$ of length $K_{max}$, the loss is computed as Equation (8).

(8) $$L_{action}=\frac{1}{K_{max}\times A}\sum_{i=1}^{K_{max}}\sum_{j=1}^{A}{\left({\hat{a}}_{i,j}-a_{i,j}^{*}\right)}^{2}$$

where ${\hat{a}}_{i,j}$ denotes the predicted action value at the i-th timestep and j-th dimension, and $a_{i,j}^{*}$ represents the corresponding ground-truth action value from the expert demonstration. This loss ensures the model learns to accurately predict the precise motor commands for the entire horizon. We formulate the prediction of the optimal chunk length $K_{t}$ as a multi-class classification problem. The ground-truth chunk length label $K_{true}$ for each training sample is generated using the heuristic algorithm described in Section 3.3.3. The loss is the cross-entropy loss between the predicted probability distribution *p* and the one-hot encoded target $K_{true}$, which is given by Equation (9).

(9) $$L_{chunk}=-\log\left(p[K_{true}]\right)$$

This loss trains the chunk size head to correctly identify the most appropriate planning horizon for a given visual context. The model is implemented in PyTorch 2.5.0 and optimized using the AdamW optimizer with a learning rate of 3 × 10^−4^ and a weight decay of 0.1. We use a batch size of 64 and train the model for 100 epochs on a dataset of 200 human demonstrations. These hyperparameters are determined based on the characteristics of the proposed dual-branch network and common practice in visual imitation learning for robotic manipulation tasks. The learning rate of 3 × 10^−4^ balances the training speed and stability, avoiding too fast convergence that may lead to overfitting or too slow convergence that results in insufficient model training. The weight decay of 0.1 is adopted to alleviate overfitting by regularizing the model parameters. The batch size of 64 is selected to ensure sufficient gradient estimation while matching the computational capacity of the training hardware (a single NVIDIA RTX 4090 GPU, NVIDIA Corporation, Santa Clara, California, USA.). The 100 training epochs are determined to ensure the model fully converges without unnecessary computational overhead. All these hyperparameters work together to guarantee the stable training of the model and the reliability of the experimental results. The complete inference procedure of the trained adaptive action chunking policy is summarized in Algorithm 1.
**Algorithm 1:** Adaptive Action Chunking Policy**Input:** Set: Trained policy parameters θ; allowable chunk set K; max steps $T_{max}$.**Initialize:** Obtain initial observation $o_{0}$, set t=0.**While** task not successful and **do****Encode:** Encode current multi-view obs. $o_{t}$: $f_{t}=E_{\theta }(o_{t})$.**Predict in Parallel:** Process with two heads:
Action Head: Predict action seq. $A_{t}=[a_{t},\ldots a_{t+K_{max}-1}]$.Chunk Head: Predict distribution $P(K_{t}|o_{t})$, set $K_{t}=\arg\max P(K_{t}|o_{t})$, where $K_{t}\in K$.**Action Selection:** Slice the first actions from $A_{t}$: $A_{t}=[a_{t},\ldots a_{t+K_{t}-1}]$.**Open-loop Execution:** Execute the actions on the robot without replanning.**Update:** $t\leftarrow t+K_{t}$. Obtain new observation $o_{t}$ after execution.**End While**

## 4. Experiments

### 4.1. Experimental Setup

To rigorously validate the superiority of the adaptive action chunking mechanism in complex manipulation tasks, we designed a series of experiments on a real-world bimanual robot platform. This section clarifies the evaluation framework shared by all experiments, including the hardware platform, baseline methods for comparison, evaluation metrics, and training details. This ensures that all performance comparisons are conducted under fair and consistent conditions.

#### 4.1.1. Hardware Platform

**Figure 2:**
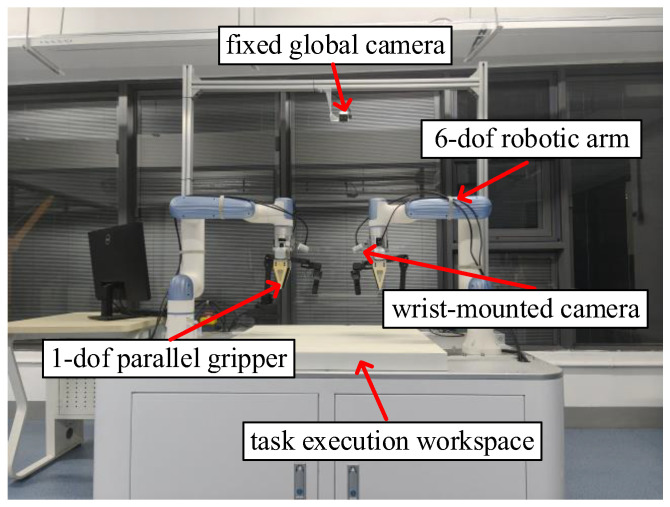
Bimanual robot system.

This work employs the open-source ALOHA bimanual robot system. As shown in Figure 2, the system is equipped with two 6-degree-of-freedom desktop robot arms, each end-effector fitted with a single-degree-of-freedom parallel gripper. The perception system consists of three RGB cameras: one global camera fixed atop the frame provides an overhead panoramic view of the scene; the other two wrist-mounted cameras are attached to the left and right arms, respectively, providing precise hand-eye visual feedback. This hardware configuration offers an ideal physical testbed for evaluating bimanual coordination and dexterous manipulation tasks.

#### 4.1.2. Baseline Methods

To systematically verify the value of the adaptive mechanism, we designed two sets of comparative experiments. The first set aims to compare against baseline methods with fixed planning horizons. We implemented three variants of the original Action Chunking Transformer, fixing their action chunk lengths to 10, 30, and 50 steps, respectively. These variants represent different static trade-offs between motion efficiency and operational precision, forming the foundation for evaluating the performance of our dynamic method.

The second set consists of an ablation study designed to isolate the contribution of the adaptive decision-making module itself. We introduced an ablation variant named Ours-Frozen. This variant is entirely consistent with the full model in terms of network architecture and input/output dimensions, but the output of its chunk size prediction head is forced to a fixed value of 25, thereby removing the adaptive capability. By comparing the performance of the full model and the ablation variant, the gain brought by the adaptive mechanism can be directly quantified.

#### 4.1.3. Evaluation Metrics

We employ task success rate and average completion time as the core evaluation metrics. The task success rate, calculated based on 20 trials with randomized initializations, objectively measures the absolute reliability of a policy. The average completion time, calculated only over successful trials, is used to reflect the policy’s execution efficiency. Together, these two metrics provide a comprehensive characterization of a policy’s overall performance.

#### 4.1.4. Training Details

All compared methods were trained under identical conditions to control for extraneous variables. Specifically, all models used the same dataset containing 200 expert demonstration trajectories, along with the same optimizer, learning rate, weight decay, batch size, and number of training epochs. The optimizer used is AdamW, with a learning rate of 3 × 10^−4^, a weight decay of 0.1, a batch size of 64, and training conducted for 100 epochs. In our proposed adaptive method, the set of allowable chunk lengths K takes the discrete values {5, 10, 15, 20, 25, 30}. This consistency ensures that any observed performance differences are attributable to the method design itself, rather than to random variations in the training process.

#### 4.1.5. Generalization Validation Scheme

To demonstrate the generalization capability of the proposed adaptive action chunking framework, a feasible verification scheme for cross-task generalization is proposed, which can be implemented based on the existing experimental platform and model architecture without additional hardware modification or model reconstruction.

Specifically, for other robotic manipulation tasks (e.g., single-arm grasping, object stacking, and precision assembly), the proposed framework can be directly reused with minor task-specific adjustments, following the verification process outlined below: First, collect expert demonstration datasets for the new target task, which follow the same data format as the existing transport-and-place and flip-and-handover tasks (including multi-view RGB images and corresponding expert action sequences). Second, fine-tune the shared Vision Transformer encoder and dual-branch prediction heads on the new demonstration dataset, while keeping the core hyperparameters (λ = 1.0, $K_{max}$ = 30, $K_{min}$ = 5, learning rate = 3 × 10^−4^, etc.) consistent with the original settings—only the final output layer of the action prediction head is adjusted to match the action space dimension of the new task. Third, conduct validation tests on the ALOHA bimanual robot platform (or a single-arm robot platform for single-arm tasks), using the same evaluation metrics as the original experiments—including task success rate, average completion time, and motion smoothness—to quantify the framework’s performance on the new task. Finally, compare the performance with fixed-chunk baselines under the same test conditions to verify the superiority of the adaptive action chunking mechanism in cross-task scenarios.

This verification scheme ensures that the proposed framework can be flexibly extended to various robotic manipulation tasks, leveraging its adaptive decision-making mechanism to maintain stable performance across different scenarios, thereby demonstrating the model’s strong generalization capability.

### 4.2. Experiment 1: Bimanual Transport-And-Place Task

#### 4.2.1. Task Description

**Figure 3:**
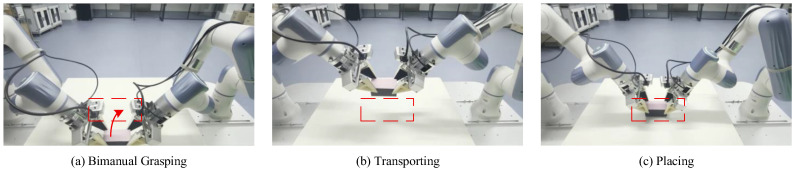
Key phases of the bimanual transport-and-place task. (**a**) Bimanual Grasping: Both arms coordinately grasp the cube; (**b**) Transporting: Both arms cooperatively move the cube toward the goal area (dashed rectangle); (**c**) Placing: The cube is stably released inside the goal area.

This experiment evaluates the proposed method on a bimanual transport-and-place task, which is designed to naturally combine the need for both long-range efficient motion and short-range precise manipulation. As shown in Figure 3, the robot must coordinate both arms to pick up a cube, transport it over a considerable distance, and place it precisely inside a marked goal area. The task is decomposed into three sequential phases: (1) bimanual grasping of the cube, (2) coordinated bimanual transport of the cube to a location above the goal, and (3) precision bimanual placement to stably release the cube inside the goal. Success is strictly defined as the cube coming to rest completely within the goal boundary. This task provides an ideal testbed for adaptive action chunking, as it presents a clear trade-off: a long planning horizon is desirable for efficient and smooth transport, while a short horizon is crucial for the accuracy and stability of the final placement.

#### 4.2.2. Quantitative Results and Analysis

**Figure 4:**
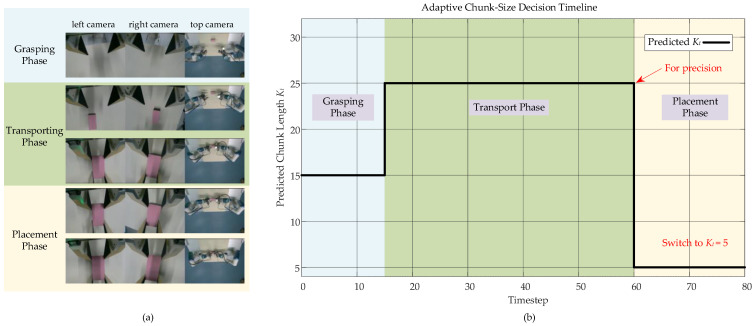
Experimental scenario of the transport-and-place task. (**a**) Image captured by the camera during the experiment; (**b**) adaptive chunk-size decision process.

To gain deeper insight into the decision-making process of the adaptive mechanism, we visualize the evolution of the predicted chunk length $K_{t}$ during a representative successful trial, as shown in Figure 4. The model exhibits a clear, phase-aware strategy. During the grasping phase, the policy selects a medium chunk length ($K_{t}=15$) for initial contact and stabilization. Upon transitioning into the long-range transport phase, it switches to and consistently selects the largest allowable chunk length ($K_{t}=25$). This long-horizon planning enables efficient and smooth motion across the workspace. A decisive switch occurs upon entering the final precision placement phase: the policy automatically and rapidly reduces the chunk length to the minimum value ($K_{t}=5$). This shift to short-horizon execution facilitates high-frequency, closed-loop adjustments, which are critical for the delicate alignment and release of the object. The step-like transition of $K_{t}$ from 15 to 25 and then to 5 clearly supports our hypothesis. The model learns to dynamically adjust its planning horizon according to task phases. This three-phase adaptive capability allows a single policy to seamlessly coordinate the full spectrum of demands, from initial stabilization and efficient long-range transport to final precise manipulation.

#### 4.2.3. Quantitative Results and Comparison

To evaluate the performance of each method, we conducted N=20 independent trials with randomized initial conditions for each. Their quantitative performance is summarized in Table 2. Our full adaptive method achieves the highest task success rate (100%) and the shortest average completion time (32.2 ± 2.3 s), achieving the best performance trade-off.

The results for the fixed-chunk baselines (ACT−K=10, 30, 50) starkly illustrate the inherent trade-off. ACT−K=10 suffers from the lowest success rate (35%), as its myopic planning leads to unstable, jittery motion during transport. Conversely, ACT−K=50 improves on success (50%) but is hampered by its inability to make fine corrections during placement, often resulting in placement failures or lengthy corrective actions, reflected in its prolonged average completion time (37.2 ± 3.8 s). ACT−K=30 finds a middle ground, achieving the best performance among fixed baselines with a success rate of 70% and a completion time of 34.8 ± 3.1 s. Nevertheless, its performance is statistically inferior to our adaptive method.

The ablation study provides conclusive evidence for the source of improvement. The Ours-Frozen variant, which uses an identical network but a fixed chunk length of K=25, achieves a success rate of 80%. This result is superior to all fixed-chunk baselines, confirming the benefit of our architectural design, yet it is consistently and significantly outperformed by the full adaptive model. This performance gap definitively attributes the principal advantage to the adaptive decision-making capability itself, rather than to any incidental increase in model capacity.

### 4.3. Experiment 2: Bimanual Alternating Flip-And-Handover Task

#### 4.3.1. Task Description

**Figure 5:**
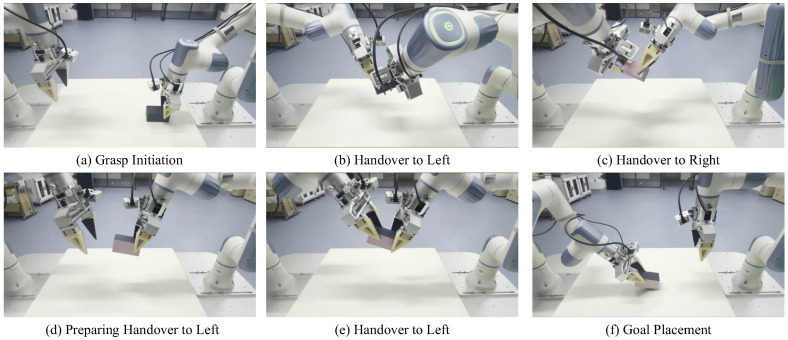
Sequential keyframes of the bimanual alternating flip-and-handover task. The sequence demonstrates the complete 180° object reorientation through multiple coordinated handovers: (**a**) initial grasp; (**b**) first handover (right to left); (**c**) second handover (left to right) completing the flip; (**d**) preparation for the final handover; (**e**) execution of the final handover, and (**f**) goal placement.

This experiment features a bimanual alternating flipping-and-handover task designed to validate the method’s adaptability in high-dynamic contact and under strict pose constraints. As shown in Figure 5, a custom cuboid with a high-contrast pink marker on one face is used. Initially, the marked face points forward. The robot must execute a sequence of precise bimanual grasps, handovers, and releases to eventually place the cuboid at the table center with its marked face rotated precisely to the backward direction. The specific sequence consists of five steps: first, the right arm grasps the object; second, it hands the object over to the left arm; third, the left arm hands it back to the right arm, which completes a 180° rotation of the object; fourth, the right arm performs a final handover to the left arm; and fifth, the left arm places the object at the goal. Success requires meeting dual criteria: the object must be stably placed within the goal area, and the orientation error of its marked face relative to the target backward direction must remain below a predefined threshold. This task critically tests the policy’s capability in instantaneous contact dynamics, bimanual coordination timing, and long-horizon pose planning.

#### 4.3.2. Quantitative Results and Analysis

**Figure 6:**
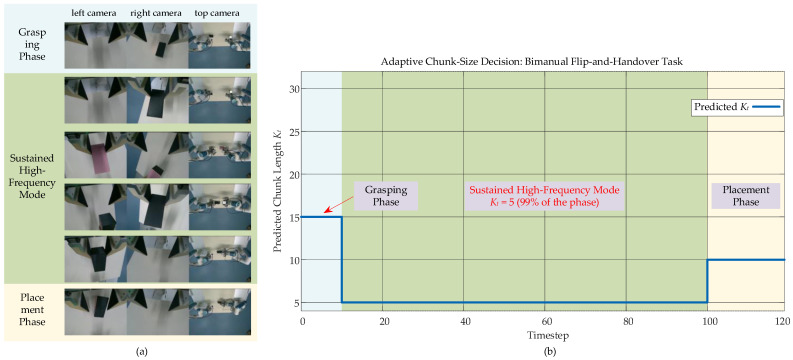
Experimental scenario of the alternating flip-and-handover task. (**a**) Image captured by the camera during the experiment; (**b**) adaptive chunk-size decision process.

In contrast to the phase-switching pattern observed in Experiment I, the adaptive mechanism exhibits a fundamentally different strategy for the highly dynamic flip-and-handover task. Analyzing the chunk length decisions from a representative successful trial, visualized in Figure 6, shows that the model persistently selects the minimum allowable chunk length, maintaining $K_{t}$ consistently at the value of 5 throughout the entire contact-rich core manipulation phase. This phase encompasses multiple handovers and the object flip. The resulting decision pattern forms a plateau-like curve near the bottom of the plot, presenting a stark visual contrast to the peak-and-valley switching pattern seen in Figure 4. This behavior shows a learned physical intuition. The model recognizes scenarios with sustained high uncertainty. Such scenarios include transient contact, slip risk, and strict alignment needs. For such scenarios, the most robust strategy is to forgo the efficiency gains of long-horizon planning and instead prioritize maximum control frequency and sensitivity. The policy’s sustained use of the minimal chunk length directly implements this strategic choice. This sustained high-frequency mode is not a pre-programmed routine but rather an endogenous decision arising from the model’s assessment of task uncertainty based on real-time visual context. This finding demonstrates that the proposed adaptive framework is capable not only of executing clear phased temporal planning but also of generating a stable and conservative control strategy for a single, prolonged, and highly challenging operational phase. Consequently, it effectively extends the framework’s general capability to handle complex manipulation scenarios.

#### 4.3.3. Quantitative Results and Comparison

To systematically evaluate the performance of each method on this highly challenging task, we conducted twenty independent trials with randomized initial conditions per method. The quantitative results are summarized in Table 3.

On this demanding task, our full adaptive method achieved a dominant success rate of 90%. In contrast, all fixed-chunk baselines performed poorly, with the most effective among them, ACT−K=30, attaining only a 25% success rate. This reveals a key limitation of fixed methods. Their performance depends on static settings, not on chunk length. They cannot adapt to real-time task changes. For instance, ACT−K=10 is forced to use the same, overly short horizon throughout the entire task, rendering it inefficient and unstable in coordinating the multi-step handovers. Its few successful trials required a lengthy 48.5 s, yet its overall success rate was merely 15%. The low success rates of 25% for ACT−K=30 and 20% for ACT−K=50 further confirm the general failure of fixed strategies when confronted with such sustained uncertainty.

The ablation variant Ours-Frozen achieved a success rate of 40%. This result, while being 1.6 times that of the best fixed baseline and confirming the value of the model architecture itself, shows a decline compared to its performance in Task 1 and remains significantly lower than that of the full adaptive model. This again demonstrates that the adaptive decision-making mechanism itself is the core enabler of performance when task uncertainty peaks. Specifically, the full model’s high success rate of 90% and its most stable completion time of 50.6 s among successful trials are directly attributable to its ability to dynamically engage the sustained high-frequency mode. In stark contrast, the lack of this capability in Ours-Frozen results in an absolute performance gap exceeding 40 percentage points.

### 4.4. Analysis and Discussion

#### 4.4.1. Summary of Core Findings

This section validates the proposed adaptive chunking framework via two robotic manipulation tasks. The two tasks have complementary properties. In the bimanual transport-and-place task, the method successfully integrates the efficiency required for long-range motion with the precision demanded by end-point operation by dynamically adjusting its planning horizon, thereby surpassing all fixed-chunk baselines across performance metrics. In the more challenging bimanual alternating flip-and-handover task, which involves sustained high-uncertainty contact, the method exhibits a completely different sustained high-frequency adjustment mode and likewise achieves superior performance compared to all baseline methods. Together, the two experiments demonstrate that the adaptive action chunking mechanism is not a technique tailored to a specific task, but a general framework capable of generating appropriate planning strategies in response to the uncertainty inherent in a task’s structure.

To account for the uncertainty and variability inherent in real-world application scenarios and to further validate the system’s anti-interference capability and environmental adaptability, we conducted targeted robustness tests based on the transport-and-place task. The entire test retained the original hardware platform, model parameters, and evaluation criteria of the existing experiment, with only reasonable perturbations applied to the experimental environment to ensure the reliability and comparability of the test results. The specific test details and outcomes are as follows.

First, the core objective of the robustness test is to verify the system’s ability to maintain stable operational performance when environmental conditions change. Therefore, drawing on the practical application scenarios of the transport-and-place task, we set up two typical types of environmental variation, which represent common interference scenarios encountered in actual operations: (1) Perturbation of the object’s initial position and orientation: On the basis of the original transport-and-place task, the initial position of the target object was randomly translated by ±5 cm, and its orientation was randomly rotated by ±10° to simulate common disturbances such as placement errors of objects or manual misalignment in real-world scenarios. (2) Visual observation noise interference: Gaussian noise with a variance of 0.01 was added to the RGB images captured by the global camera and the wrist-mounted cameras. This simulates visual perception deviations caused by lighting changes, camera lens contamination, or minor equipment interference in practical applications, reflecting the complexity of real operating environments.

Second, the test procedure strictly followed the original experimental specifications to ensure reproducibility. For each environmental variation condition, both our method and all fixed-chunk baseline methods were tested 20 times. For each test, we recorded the task completion status, the average completion time, and the trajectory deviation. The criterion for task success remained consistent with the original experiment, meaning the object was successfully transported from the initial area to the target area without being dropped or exceeding the allowable position deviation.

Finally, the test results fully validated the strong robustness of the proposed system. Under the two environmental variation conditions, our adaptive chunking method, leveraging its advantage of dynamically adjusting the planning horizon, maintained a task success rate of over 90% stably. In contrast, the success rate of the fixed-chunk baseline methods dropped to only 30–60%. The specific test data are summarized in Table 4.

The above robustness test results are consistent with the core experimental results, which not only further consolidate the advantages of the proposed method in conventional scenarios but also clearly prove that the method can effectively cope with environmental changes in practical applications. It enhances the system’s anti-interference capability through an adaptive adjustment strategy, solves the problem of a significant performance degradation of fixed-chunk methods under environmental changes, and provides strong experimental support for the practical application of the proposed method.

#### 4.4.2. Multi-Modality of the Adaptive Strategy

A key finding of this work is that the adaptive mechanism can generate two functionally distinct yet equally intelligent decision-making patterns. In Experiment 1, the strategy manifests as a clear phase-aware switching behavior. Specifically, it proactively selects a long horizon during the open transport phase to ensure motion efficiency and then switches to a short horizon at the critical placement phase, requiring fine manipulation, to achieve high-precision control. This behavioral pattern aligns with the prior phased structure of the task itself. In stark contrast, the strategy in Experiment 2 presents a sustained high-frequency execution mode. Throughout the core manipulation phase, rich in dynamic contact, the model almost consistently adopts the minimum chunk length. This decision is not because the task is predefined as a high-frequency phase but stems from the model’s assessment during real-time execution that the entire continuous contact sequence constitutes a situational unit of sustained high uncertainty, thereby leading to the proactive choice of the most conservative yet reliable high-frequency adjustment strategy.

These two modes show that our method learns more than a simple scene-to-chunk mapping. It learns a general principle: choosing the planning horizon by evaluating uncertainty. When uncertainty is temporally concentrated and predictable, such as the placement moment in Experiment 1, the strategy performs precise phase switching. When uncertainty is temporally diffuse and persistent, such as the entire handover process in Experiment 2, the strategy stabilizes in a globally conservative mode. This ability to dynamically derive different strategies based on real-time perception is unattainable by any fixed strategy or predefined two-stage heuristic method. It also highlights the powerful advantage conferred by combining end-to-end learning with an adaptive decision-making architecture.

#### 4.4.3. Limitations and Future Work

Despite the promising results of this study, the current framework has certain limitations that also point to directions for future research. First, the performance ceiling of our method is constrained by the quality and coverage of the expert demonstration data. Its generalization capability may face challenges in completely unseen scenarios that require creative solutions. Second, the current chunk length decision is purely based on visual observation at the current timestep, constituting a reactive strategy that lacks explicit proactive reasoning about long-term task progression. Additionally, the current method does not explicitly account for the influence of noise in robotic systems, such as visual perception noise, actuator noise, and environmental disturbance, which are common in practical robotic applications and have been emphasized as a crucial factor for practical deployment in recent related studies [32,33]. Furthermore, the adaptive mechanism introduced in this work adds additional prediction and decision steps, which may bring potential impacts on inference latency and scalability, and these aspects need further discussion. In addition, the current framework does not focus on capturing the operator’s preferences, which is an interesting extension question regarding whether imitation learning can partially capture such preferences, as recent studies have explored preference-aware robotic systems (e.g., using surgeon preferences to realize autonomous instrument tracking) [34].

Addressing the above limitations, future work could explore several promising directions. The first direction is to investigate integrating the low-level adaptive execution layer proposed in this work with a high-level task planner, for instance, a large language model. The high-level planner could provide task semantic decomposition and sub-goal sequences, thereby offering proactive guidance for chunk length prediction and enabling decisions that serve long-term task intent beyond merely reacting to the immediate state. The second direction involves combining the current framework with online reinforcement learning paradigms. This would allow robots to autonomously explore and optimize chunk length selection strategies for different contexts through interaction with the environment, thereby reducing reliance on extensive demonstration data and enabling autonomous skill acquisition. The third direction is to research more efficient adaptive decision-making network architectures to reduce computational overhead and to explore their deployment and generalization on more complex platforms such as humanoid robots or mobile manipulators. The fourth direction is to further enhance the noise robustness of the proposed adaptive action chunking framework. Specifically, we will add noise suppression modules to the visual encoder. This reduces the influence of visual noise on feature extraction and chunk-length prediction. Additionally, we will investigate the robustness of the dual-branch prediction heads to actuator noise and explore adaptive adjustment strategies that can dynamically compensate for noise-induced errors, which will further improve the practicality and reliability of the proposed framework in complex real-world scenarios with noise interference. The fifth direction is to discuss and optimize the inference latency and scalability of the framework. For inference latency, we will explore lightweight network design strategies for the dual-branch prediction heads to reduce the computational cost of additional prediction and decision steps, ensuring the framework can meet the real-time requirements of robotic manipulation tasks. For scalability, we will further study the adaptability of the framework to different robot platforms and task scales, optimizing the network structure to enable flexible deployment in scenarios with varying computational resources and task complexities. The sixth direction is to explore the possibility of capturing the operator’s preferences through imitation learning. Building on recent research on preference-aware robotic systems, we will investigate how to incorporate operator preference information into the adaptive action chunking framework, for example, by analyzing the characteristics of expert demonstration data to extract implicit preference patterns, which will further enhance the adaptability and practicality of the framework to human–robot interaction scenarios.

## 5. Conclusions

Robot imitation learning has long faced a trade-off. Fixed action chunking must choose either motion efficiency or manipulation precision. This paper presents a novel adaptive action chunking framework designed to fundamentally address this issue. The core idea is to move beyond a static planning horizon and instead train robots to dynamically predict the optimal action chunk length for each step based on real-time visual context. To this end, we designed an end-to-end dual-branch neural network. A shared encoder fuses multi-view visual inputs, while parallel prediction heads generate future action sequences and optimal chunk length decisions, respectively. Systematic experiments on a real-world bimanual robot platform demonstrate the framework’s exceptional performance across two distinct classes of complex manipulation tasks. It not only achieves intelligent phase-aware switching in tasks with clear sequential structure but also reliably adopts a sustained high-frequency adjustment mode for dynamic contact tasks filled with persistent uncertainty. These results collectively affirm that adaptive planning is key to overcoming the inherent limitations of fixed strategies and achieving general dexterous manipulation.

The contributions of this work are threefold: First, it proposes a new paradigm of adaptive action chunking. This work is the first to unify long-horizon action generation and dynamic planning-horizon decision-making within a single end-to-end imitation learning framework, theoretically overcoming the limitations of fixed-chunk strategies and providing a new technical pathway for robots to flexibly meet diverse task demands. Second, it designs and validates an efficient dual-branch network architecture. Through the synergy of a visual encoder and parallel prediction heads, this architecture enables the joint optimization of action prediction and chunk length decisions. Systematic ablation studies confirm that the performance gain stems from the adaptive decision-making mechanism itself, not merely increased model capacity. The design proves to be both effective and trainable. Third, it conducts comprehensive and rigorous empirical research. Through two meticulously designed tasks—“bimanual transport-and-place” and “bimanual alternating flip-and-handover”—this work provides ample quantitative and qualitative evidence. The experiments not only demonstrate that the proposed method significantly outperforms existing baselines in both success rate and efficiency but also, through in-depth visualization of the decision process, reveal that the adaptive mechanism can generate distinctly different intelligent strategic patterns in response to the uncertainty structure of tasks. This offers valuable insights for understanding the behavior of such models.

This work opens several promising directions for dexterous robot manipulation. For algorithm improvement, future work can integrate adaptive chunking with high-level task planners. For instance, incorporating large language models for semantic understanding and task decomposition could provide proactive guidance for chunk length decisions, enabling them to serve more complex long-horizon instructions. In terms of learning paradigms, combining this framework with reinforcement learning holds the potential for robots to continuously optimize their chunking strategies through autonomous interaction with the environment, facilitating a leap from imitation learning to autonomous skill discovery. Regarding system application, validating the framework’s generalization capability across different robot morphologies and a broader spectrum of task domains, particularly its adaptability on complex systems like humanoid robots and mobile manipulators, will be crucial steps toward practical deployment. These explorations will not only deepen the understanding of adaptive decision-making mechanisms but also lay a solid technical foundation for building truly intelligent and general-purpose robotic systems.

## Figures and Tables

**Table 1:** Comparison of the Proposed Method with Previous Representative Approaches.

Approach	Core Framework	Chunk Length Type	Bimanual Manipulation Adaptation
ACT [5]	Action Chunking Transformer	Fixed (predefined)	not support
Diffusion-based ACT [21]	Action Chunking + Diffusion Model	Fixed (predefined)	not support
Hierarchical Skill Learning [27]	Hierarchical Planning + Skill Library	Fixed (skill-specific)	support
Meta-learning-based Adaptation [31]	Meta-learning + Parameter Adaptation	no action chunking	not support
Autoregressive Action Learning [24]	Autoregressive Sequence Prediction	Fixed (predefined)	not support
Proposed Method (Ours)	Adaptive Action Chunking + Dual-branch Network	Dynamic (context-aware)	support

**Table 2:** Performance comparison on the bimanual transport-and-place task.

Method	Success Rate (%)	Average Completion Time (s)
ACT−K=10	35	38.5 ± 4.2
ACT−K=30	70	34.8 ± 3.1
ACT−K=50	50	37.2 ± 3.8
Ours-Frozen (K=25)	80	35.5 ± 2.9
Ours (Adaptive)	100	32.2 ± 2.3

**Table 3:** Performance comparison on the bimanual alternating flip-and-handover task.

Method	Success Rate (%)	Average Completion Time (s)
ACT−K=10	15	48.5 ± 7.2
ACT−K=30	25	48.1 ± 9.5
ACT−K=50	20	51.5 ± 10.3
Ours-Frozen (K=25)	40	45.5 ± 7.9
Ours (Adaptive)	90	50.6 ± 6.2

**Table 4:** Robustness Test Results of the Transport-and-Place Task.

Test Scenario	Proposed Method (Success Rate)	Fixed Chunk (*K* = 10, Success Rate)	Fixed Chunk (*K* = 30, Success Rate)
Normal Scenario (No Interference)	100%	35%	70%
Object Position/ Orientation Perturbation	100%	35%	60%
Visual Noise Interference	95%	30%	50%

## Data Availability

The original contributions presented in this study are included in the article. Further inquiries can be directed to the corresponding author.
